# Supplementary material for: Asthma status and suicidal behavior risk: A meta-analysis of cohort studies
Source: PLoS One. 2025 Jun 3;20(6):e0325150. doi: 10.1371/journal.pone.0325150 (PMC12133172; doi:10.1371/journal.pone.0325150)
Supplement: S1 File — (DOCX) [file pone.0325150.s001.docx]

Table 1: PubMed 2025.01.05

| Search number | Query | Results |
| --- | --- | --- |
| 1 | "Asthma"[Mesh] | 147,556 |
| 2 | Asthma*[Title/Abstract] | 192,167 |
| 3 | ("Asthma"[Mesh]) OR (Asthma*[Title/Abstract]) | 212,335 |
| 4 | "Suicide"[Mesh] | 79,194 |
| 5 | Suicide*[Title] | 41,477 |
| 6 | ("Suicide"[Mesh]) OR (Suicide*[Title]) | 85,922 |
| 7 | (("Asthma"[Mesh]) OR (Asthma*[Title/Abstract])) AND (("Suicide"[Mesh]) OR (Suicide*[Title])) | 132 |

Table 2: Embase 2025.01.05

| No. | Query | Results |
| --- | --- | --- |
| #1 | 'asthma'/exp | 334361 |
| #2 | 'asthma*':ab,ti | 284928 |
| #3 | #1 OR #2 | 373935 |
| #4 | 'suicide'/exp | 75146 |
| #5 | 'suicide*':ab,ti | 104383 |
| #6 | #4 OR #5 | 127085 |
| #7 | #3 AND #6 | 548 |

Table 3: Cochrane Library 2025.01.05

| ID | Search | Hits |
| --- | --- | --- |
| #1 | MeSH descriptor: [Asthma] explode all trees | 15214 |
| #2 | (Asthma*):ti,ab,kw (Word variations have been searched) | 39229 |
| #3 | MeSH descriptor: [Suicide] explode all trees | 2270 |
| #4 | (Suicide*):ti,ab,kw (Word variations have been searched) | 6091 |
| #5 | #1 OR #2 | 39229 |
| #6 | #3 OR #4 | 6354 |
| #7 | #5 AND #6 | 41 |

Figure 1 Sensitivity analysis of current asthma and suicidal behavior risk

Figure 2 Funnel plot of current asthma and suicidal behavior risk

Figure 3 Sensitivity analysis of asthma history and suicidal behavior risk

Figure 4 Funnel plot of asthma history and suicidal behavior risk
